# Development and Implementation of an IoT-Enabled Optimal and Predictive Lighting Control Strategy in Greenhouses

**DOI:** 10.3390/plants10122652

**Published:** 2021-12-02

**Authors:** Shirin Afzali, Sahand Mosharafian, Marc W. van Iersel, Javad Mohammadpour Velni

**Affiliations:** 1 School of Electrical and Computer Engineering, University of Georgia, 111 Boyd Graduate Studies Research Center, 200 D.W. Brooks Drive, Athens, GA 30602, USA; shirin.afzali@uga.edu (S.A.); sahandmosharafian@uga.edu (S.M.); 2 Department of Horticulture, University of Georgia, 1111 Miller Plant Sciences Building, Athens, GA 30602, USA; mvanier@uga.edu

**Keywords:** Internet of Things (IoT), optimal control, supplemental lighting in greenhouses, image processing

## Abstract

Global population growth has increased food production challenges and pushed agricultural systems to deploy the Internet of Things (IoT) instead of using conventional approaches. Controlling the environmental parameters, including light, in greenhouses increases the crop yield; nonetheless, the electricity cost of supplemental lighting can be high, and hence, the importance of applying cost-effective lighting methods arises. In this research paper, a new optimal supplemental lighting approach was developed and implemented in a research greenhouse by adopting IoT technology. The proposed approach minimizes electricity cost by leveraging a Markov-based sunlight prediction, plant light needs, and a variable electricity price profile. Two experimental studies were conducted inside a greenhouse with “Green Towers” lettuce *(Lactuca sativa)* during winter and spring in Athens, GA, USA. The experimental results showed that compared to a heuristic method that provides light to reach a predetermined threshold at each time step, our strategy reduced the cost by 4.16% and 33.85% during the winter and spring study, respectively. A paired t-test was performed on the growth parameter measurements; it was determined that the two methods did not have different results in terms of growth. In conclusion, the proposed lighting approach reduced electricity cost while maintaining crop growth.

## 1. Introduction

The global population is predicted to grow to around 9.15 billion by 2050, which will increase the amount of food that needs to be produced [1]. Furthermore, the limitations of natural resources and productive land, as well as climate constraints raise concerns about food security. The rising demand for food has attracted researchers’ attention towards the application of Internet of Things (IoT) technology in agriculture. The IoT is a network of physical objects that transfer data to other devices over the Internet [2]. Applying the IoT in controlled environment agriculture (CEA) has reduced human effort, time, and cost and resulted in yield improvements [3]. The IoT integrates several technologies such as wireless sensor networks (WSNs), radio-frequency identification (RFID), cloud and edge computing, and human–computer interaction (HCI) [4].

The application of the IoT in agriculture includes monitoring, control of agriculture machinery, tracking and tracing, precision agriculture, and greenhouse production [3]. Monitoring and acquiring data about some environmental factors in crop farming such as temperature, humidity, solar radiation, pest movement, and rainfall help to understand the patterns and maximize farm production [5]. Other than crop farming, monitoring the water quality, water level, and temperature levels in aquaponics is another application of the IoT [6]. Furthermore, factors to be monitored in forestry [7] and livestock [8] are other applications of the IoT in agriculture. Although some strategies have been developed in the area of monitoring, developing cost-effective methods is still an open area [3]. Both manned and autonomous vehicles (such as unmanned aerial vehicles (UAVs)) can collect useful data for farmers; they can also be remotely controlled through the IoT system [9]. Applying the IoT in tracking and tracing can improve agricultural companies’ supply chain. Tracking is related to capturing, collecting, and storing data along the supply chain from upstream to downstream, while tracing enables distinguishing the product from downstream to upstream [10].

Another application of the IoT is in precision agriculture, that is a management strategy that collects real-time data such as crop maturity, weather, air quality, etc., and then analyzing the data to improve crop yields and reduce cost [11]. IoT technology could be employed in greenhouses to maximize profit, reduce cost and labor, and save energy. Several studies have considered applying WSNs in greenhouses for monitoring [12,13,14]. In this study, we focus on the application of IoT technology in greenhouse production.

Supplemental lighting improves plant growth and contributes to higher yields in CEA, in particular greenhouses. During rainy days or winter months, the overall amount of sunlight that plants receive might not be sufficient for plant growth and development. Therefore, supplemental lighting is often necessary for greenhouse fruit and vegetable production. However, the electricity needed for greenhouse supplemental lighting amounts to about 30% of the operating costs [15]. As a result, enhancing the cost effectiveness of lighting with modern technologies plays an important role in the CEA industry.

Among various lighting types, light-emitting diodes (LEDs) have proven to be more effective, due to their dimming capability, which enables changing the intensity of the output light to any continuous level [16]. On the other hand, the output light of high-intensity discharge (HID) lamps only takes some quantized levels, thereby not considered as the primary choice for optimal lighting strategies. Researchers have proposed rule-based supplemental lighting control methods since 1994; the authors in [17] developed a rule-based approach for HID lamps to reach a predefined light target within a specified photoperiod. Light and shade system implementation (LASSI) is another control method with an on/off control for HID lamps in combination with a sunlight prediction and movable shades to achieve a constant DLI [18]. Another version of LASSI improved the sunlight prediction, resulting in increasing the accuracy of lighting control [19].

In [20], the DynaLight system, an on/off control method for HID lamps, was developed. This system considers a leaf photosynthesis model, variable electricity pricing, and weather forecast to reduce electricity cost while reaching a minimum daily sum of photosynthesis. Nevertheless, the weather forecasts were provided twice daily at an hourly resolution [20], which is not precise enough for proper optimization. Another version of DynaLight for controlling HID lamps is called DynaLight IND, which is a multi-objective optimization platform for optimizing artificial lighting in greenhouses [21]. For DynaLight IND, an evolution strategy was proposed and compared with the original genetic algorithm (GA) in DynaLight. The simulation results showed that the new version improved the GA’s evolution efficiency and increased the computation speed compared to the original DynaLight. However, other than simulations, there is a need to conduct practical experiments to validate the properties of DynaLight IND [21].

The adaptive lighting method [22] is another rule-based control approach that reduces cost by adjusting the duty cycle of LEDs to reach a specified threshold based on photosynthetic photon flux density (PPFD) levels. Two general approaches were taken in previous studies on supplemental lighting control with LEDs: either using real-time sunlight intensity measurements to supply fixed PPFD levels [23,24] or assuming prior information about sunlight intensity throughout the day [23]. Both of these perspectives are faulty since sunlight intensity throughout the day is unknown and using the current PPFD alone does not ensure reaching the daily light integral (DLI) or daily photosynthesis.

In our prior studies [25,26], we developed optimal supplemental lighting strategies for both LEDs and HID lamps in greenhouses, which significantly reduced the electricity cost. We formulated the supplemental lighting control problem as a constrained convex optimization problem, and we aimed at minimizing the electricity cost of supplemental lighting, while considering sunlight prediction, plant light needs, and variable electricity pricing in our model. This strategy used a Markov model to predict future sunlight intensities. The simulation studies showed that the proposed method could reduce electricity cost significantly [25]. To control the light intensity of HID lamps, we formulated a discrete constrained optimization problem, which was solved using the method of multipliers and a reinforcement learning (RL) algorithm, considering the Markov-based sunlight prediction [26]. Although we made sure to provide sufficient light for good crop growth as the constraint of the optimization problem, it is necessary to implement the method in the greenhouse and monitor plant growth through experimental studies.

In the present paper, we implemented our proposed lighting strategy for LEDs in a research greenhouse equipped with IoT technology. Lettuce was grown under two treatments with different lighting control strategies over two seasons with low and high natural light (winter and spring). To evaluate the proposed lighting control approach in terms of plant growth, we collected data related to growth during both experiments. The main contributions of this work are as follows: (1) We implemented an optimal supplemental lighting control strategy in a greenhouse equipped with IoT technology. (2) We evaluated the advantages of our proposed optimal lighting method based on not only the electricity cost, but also the plant growth through experimental studies.

## 2. Materials and Methods

### 2.1. Preliminaries and Problem Formulation

Photosynthesis is a photon-driven process. Thus, photosynthetic light levels are measured as the photosynthetic photon flux density (PPFD) with units of µmol m${}^{-2}$ s${}^{-1}$. The PPFD is the light-photon numbers in the photosynthetically active wavelength range (400–700 nm) per square meter per second. The daily light integral (DLI) in mol m${}^{-2}$ d${}^{-1}$ is the integral of the PPFDs over 24 h. To ensure sufficient growth for plants in greenhouses, it is recommended that they receive a minimum amount of DLI during the photoperiod, which is the time period each day (up to 24 h) during which plants receive light [23]. To formulate the optimization problem, the relation between two important parameters in plant photosynthesis was considered. These parameters are the PPFD and electron transport rate (ETR), which is the number of electrons transported through photosystem II per square meter of leaf area per second (with units of µmol m${}^{-2}$ s${}^{-1}$). The daily photochemical integral (DPI) in mol m${}^{-2}$ d${}^{-1}$ is the integral of the ETRs over 24 h. Furthermore, the ETR and PPFD generally have an exponential rise to a maximum relation. The authors in [23] derived a relationship between the ETR and PPFD as:

$$\begin{array}{c} ETR=a\;(1-e^{-k\times PPFD}), \end{array}$$

where *a* is the asymptote of the ETR and *k* is the initial slope of the ETR divided by *a*. For “Green Towers” lettuce, which was used in this study, a=121 µmol m${}^{-2}$ s${}^{-1}$, and k=0.00277 [23].

For many greenhouse crops, to guarantee high-quality production and adequate growth, a minimum DLI is suggested. In some cases, a specific photoperiod must also be achieved. However, the DPI and plant growth depend on the combination of the DLI and photoperiod; longer photoperiods with the same DLI result in a higher DPI and more biomass [27,28]. Therefore, the DPI is a better predictor of plant growth and better suited for lighting optimization algorithms than the DLI. “Green Towers” lettuce requires a DPI of 3 mol m${}^{-2}$ d${}^{-1}$, corresponding approximately to a DLI of 17 mol m${}^{-2}$ d${}^{-1}$ under ambient sunlight conditions [23]. The optimization problem was formulated to minimize the total amount of supplemental lighting cost to reach a specified DPI within a specified photoperiod.

The theory behind the experiments considered in this study is based on the constrained nonlinear optimization problem presented in [25], which is as follows:

(1) $$\begin{array}{c} \min_{\bar{x}}\;f\left(\bar{x}\right)=\sum_{t=1}^{T}\frac{C_{t}}{k}\left[\ln\left(\frac{a}{a-{\bar{x}}_{t}-{\bar{s}}_{t}}\right)-s_{t}\right]\\ \mathrm{subject}\;\mathrm{to}:\;\;\sum_{t=1}^{T}\left({\bar{x}}_{t}+{\bar{s}}_{t}\right)\geq \frac{\bar{D}}{m}\\ {\bar{x}}_{t}\geq 0\;\;;\;\;t=1,2,\cdots ,T\\ {\bar{x}}_{t}\leq {\bar{U}}_{LED}\;\;;\;\;t=1,2,\cdots ,T, \end{array}$$

where ${\bar{x}}_{t}$ is the ETR resulting from supplemental light provided by the LEDs at time step *t*, ${\bar{s}}_{t}$ is the ETR resulting from sunlight, $s_{t}$ is the PPFD received from the Sun, ${\bar{U}}_{LED}$ is the maximum ETR that can be achieved with LEDs, $C_{t}$ is the electricity price in cents/kWh, $\bar{D}$ is the minimum DPI needed for the plant during the entire photoperiod, m is the length of each time step in seconds, and *T* is the number of time steps. The first constraint in (1) guarantees supplying sufficient light to the plants to reach the recommended DPI, and the other constraints define the ETR bounds according to the PPFD of LEDs.

A photoperiod of 16 h is common for greenhouse lettuce production and used to illustrate the performance of the control strategy. The optimization problem was solved at each time step (with the length of m seconds) during the allowed photoperiod for each day, and supplemental light was provided up to the optimal PPFD calculated for that time step. The process was repeated every m seconds time step, for a total number of T=16×3600/m when a 16 h photoperiod was used. The Markov-based predictive values are substituted in (1), instead of the actual future sunlight intensities (which are not obtainable in real time). For a detailed description on sunlight prediction using Markov chains, we refer to our previous work [25]. Consequently, (1) can be demonstrated as:

(2) $$\begin{array}{c} \underset{\bar{x}}{\mathrm{minimize}}\;\sum_{t=i}^{T}\frac{C_{t}}{k}\left[\ln\left(\frac{a}{a-{\bar{x}}_{t}-{\bar{s}}_{t}}\right)-s_{t}\right]\;\;\;\;\;\;\;\;\;\;\\ \mathrm{subject}\;\mathrm{to}:\;\;\sum_{t=i}^{T}\left({\bar{x}}_{t}+{\bar{s}}_{t}\right)\geq \frac{\bar{D}}{m}-\sum_{t=1}^{i-1}\left({\bar{x}}_{t}+{\bar{s}}_{t}\right),\\ {\bar{x}}_{t}\geq 0;\;\;\;\;\;\;t=i,i+1,\cdots ,T,\;\;\;\;\;\;\;\;\;\;\;\;\;\;\\ {\bar{x}}_{t}\leq {\bar{U}}_{LED};\;\;\;\;\;\;t=i,i+1,\cdots ,T.\;\;\;\;\;\; \end{array}$$

The optimization problem (2) was solved once before sunrise and once after sunset. Throughout the day, (2) was solved repeatedly at each time step. The interested reader is referred to [25] for more details on how to calculate the optimal lighting strategy.

### 2.2. IoT Structure

Four essential components of an IoT ecosystem include: (1) IoT devices; (2) communication technology; (3) the Internet; and (4) data storage and processing [3]. The description of each component is provided as follows:

(1) IoT devices: IoT devices consist of embedded systems, which include microprocessors, field programmable gate arrays (FPGAs), and input/output interfaces that interact with sensors and actuators [3]. In this study, a quantum light sensor was used to measure sunlight irradiance in the greenhouse, and an infrared (IR) night vision camera was deployed to monitor growth and calculate the projected canopy size (PCS) (a morphological indicator of plant growth). A Raspberry Pi was also used as the microprocessor, which was connected to the camera and the light sensor through an analog-to-digital converter (ADC). Data were stored on the Raspberry Pi SD card and then transferred through WiFi. Reliability, portability, security, and cost were taken into account while choosing the IoT devices;
(2) Communication technology: Communication technology in IoT systems can be categorized based on the standards, spectrum, and application scenarios. In the deployment of wireless connectivity, the range of the communication distance, data rate, security and resilience, and cost of gateway modems are some of the parameters that should be considered [3]. For our study, WiFi was chosen as the wireless technology with a WLAN and WPA2 security;
(3) Internet: The connection between IoT devices and the Internet allows transferring data towards a remote infrastructure for storage, processing, and further analysis. Security, accessibility, and support for real-time data should be taken into account while transferring data [3];
(4) Data storage and processing units: Data collected in agriculture applications can be in different forms including images, text, audio, and video. Cloud IoT platforms have been used to store big data collected from sensors. Some of the commercial platforms that provide data storage and analysis are Onfarm systems, Cropx, KAA, Farmx, Easyfarm, and Farmlogs [3]. In our experiment, data in the form of text and images were stored on the Raspberry Pi SD card and retrieved remotely using WPA2 and RealVNC software (RealVNC is available at https://www.realvnc.com/en/, accessed on 20 November 2020).

### 2.3. Experimental Setup

To evaluate the performance of the proposed lighting control approach in terms of plant growth, as well as electricity cost, two experiments were conducted inside of a research greenhouse for “Green Towers” lettuce in Athens, GA, USA. The experimental unit consisted of a group of 15 plants in 10 cm${}^{2}$ pots on each bench. Those pots were filled with a soil-less substrate (80% peat: 20% perlite (*v*/*v*) (Fafard 1P; SunGro Horticulture, Agawam, MA, USA)). Plants were grown under two supplemental lighting treatments (the proposed method and a heuristic one) and were manually irrigated every two or three days with a nutrient solution containing 100 mg L${}^{-1}$ N made with a water-soluble fertilizer (15N–2.2P–12.45K, Peters Excel 15–5–15 Cal-Mag Special; Everris NA Inc., Dublin, OH, USA).

We compared the lighting electricity cost of our proposed method to two supplemental lighting strategies: (1) baseline, which is also optimal and solves the optimization problem (2) assuming perfect prior knowledge of sunlight throughout the day; (2) heuristic, in which the goal was to supply enough supplemental light to reach a minimum PPFD, unless the PPFD from sunlight alone exceeded the threshold. Thus, by the end of photoperiod, the plants will have received enough supplemental light to reach a DPI greater than or equal to the suggested quantity. In this method, sunlight prediction and real-time electricity price are not taken into account. Since the assumption of perfect prior knowledge of sunlight is not realistic nor practical, only the heuristic method and the proposed method were implemented in the greenhouse to grow lettuce under those treatments and compare plant growth. However, the simulation results related to the electricity cost for the baseline method are provided and represent a theoretical optimal scenario.

Our control algorithms were implemented on the Raspberry Pi 3 Model B (a low-cost, small microprocessor) using the Python programming language. Raspberry Pi operates as a control hardware to decide on how much supplemental lighting the plants need based on the predicted sunlight. CVXPY, a domain-specific language (DSL) that enables solving convex optimization problems with the high-level features of Python [29,30], was used to calculate the optimal lighting (CVXPY is available at http://www.cvxpy.org, accessed on 1 January 2020).

To develop the Markov model for sunlight prediction, sunlight data collected from the NREL database [31] were taken as representative of the sunlight intensity outside our greenhouse. Often, 40–70% of the sunlight PPFD represents the PPFD measured at a leaf surface in a greenhouse because of the building materials of the greenhouse ceiling and the sunlight radiation angle [32]. For the development of the Markov model, we considered 40% as the light transmission rate since the greenhouse had an internal shade cloth, which was used to achieve low sunlight levels and facilitate lighting research. This model was used in both experiments to predict sunlight based on real-time PPFD measurements by a light sensor inside the greenhouse, underneath the shade cloth. These measurements represent the amount of sunlight that reached the plants. The parameters used in our optimization problem were similar to those considered in our prior study [25] and for the type of LEDs used in the experiments (given in Table 1).

To measure the sunlight PPFD over the plant canopy in the greenhouse, an analog full-spectrum quantum sensor (SQ-500-SS, Apogee instruments, Logan, UT, USA) with an improved spectral range of 389–692 nm ± 5 nm was used. SQ-500-SS is a self-powered PAR light sensor with a 0–40 mV output and calibration factor of 100 µmol m${}^{-2}$ s${}^{-1}$ mV. Sunlight intensity was measured by the light sensor using a Python script every three minutes, then averaged to be representative of sunlight at each time step (15 min). Since the Raspberry Pi only reads digital inputs, the analog output of the sensor was converted to digital using ADS1115, which is a high-precision 16 bit ADC. ADS1115 uses the I2C communication protocol to read analog values; therefore, I2C should be enabled on the Raspberry Pi before interfacing.

The supplemental lighting source used in our setup was the GE Arize${}^{\mathrm{TM}}$ element L1000 LED grow light bars with a 3:1 red-to-blue light ratio (HPPB4) and a power of 627 W. Several methods of controlling the output signal of LED drivers are available; the 0–10 V dimming approach was used for our purposes. In this method, there is a near-linear relationship between the dimming voltage of the LED driver and the output light of the LED. To control the output light of the LEDs, two GPIO pins of the Raspberry Pi (GPIO 13 and GPIO 18) were used to generate two pulse-width modulation (PWM) signals. One PWM signal controls the LEDs under the heuristic treatment, and the other one controls the LEDs under the prediction-based treatment. These PWM signals of the Raspberry Pi need to be amplified and filtered to become compatible with the dimming voltage of the LED driver; thus, a signal-conditioning circuit (using the TL081 operational amplifier (op-amp)) was designed to supply the proper signal to the LED drivers. Based on the calculated supplemental lighting strategy, the duty cycle of the PWM signals was determined by the Raspberry Pi and converted to the compatible dimming voltage (control signal) for LED drivers by the signal-conditioning circuit. At each time step (15 min), the control signals for the two treatments were adjusted, thereby changing the output light of the LEDs.

Every morning, the Raspberry Pi automatically runs the Python code, which reads real-time light sensor data, predicts future sunlight, solves the optimization problem, and calculates the supplemental lighting for both treatments at each time step (every 15 min) for a 16 h photoperiod. Once the photoperiod is over and plants receive sufficient light, the LEDs are turned off.

**Figure 1:**
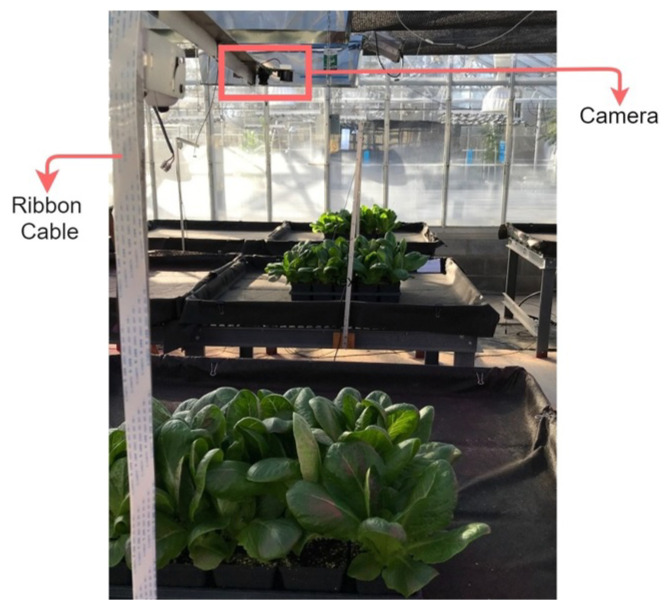
Imaging setup built in the greenhouse.

To monitor the plant growth and PCS for the two treatments, Arducam day–night vision cameras were installed above the benches. The camera was mounted facing downward above the crop. These cameras have an IR cut filter that switches automatically. The filter is on for daylight color accuracy and off for IR night vision. Therefore, the camera can take pictures at night in complete darkness using its small IR LEDs. Moreover, this camera is an inexpensive choice (less than USD 30) for monitoring plant growth. Each camera was connected to a Raspberry Pi, which was set to automatically take pictures every night when the LEDs were off, so that there would be no sunlight or LED light interfering with the plant images. Figure 1 shows the imaging setup.

We accessed the data related to supplemental lighting, sunlight, cost, and plant images through real-time remote monitoring of the Raspberry Pi using WPA2 and RealVNC software. Plant images were processed using PlantCV, which is an image analysis package for plant phenotyping [33] (PlantCV is available at https://plantcv.readthedocs.io/en/stable/, accessed on 20 November 2020). The PCS was measured by using a reference with a known area and counting the number of detected leaf pixels.

**Figure 2:**
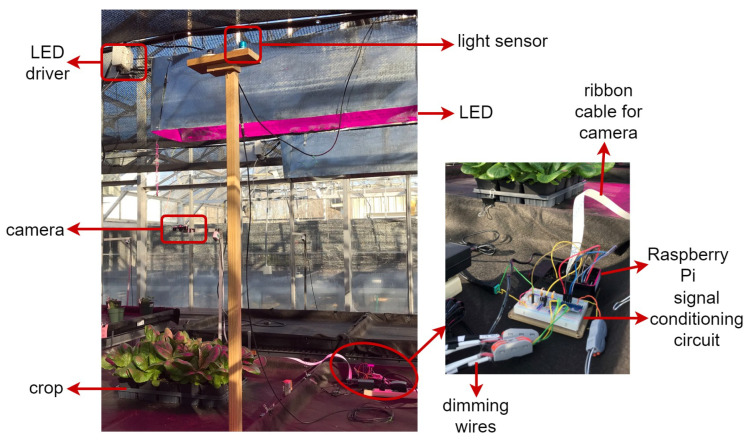
Experimental setup in the greenhouse.

Shown in Figure 2 is the experimental setup in the greenhouse. The light sensor was installed above the LED fixtures so that it was not affected by the LED light and was connected to the ADC. The ADC converts the analog measurements to digital, and hence, the Raspberry Pi can read them. After predicting sunlight and solving the optimization problem, the duty cycle of the PWM signals was determined and amplified. These control signals were connected to the dimming wires of the LED driver, thereby changing the output light of the LED. The camera was installed above the crops and connected to the Raspberry Pi through a ribbon cable. The materials used in the proposed setup are presented in Table 2.

**Figure 3:**
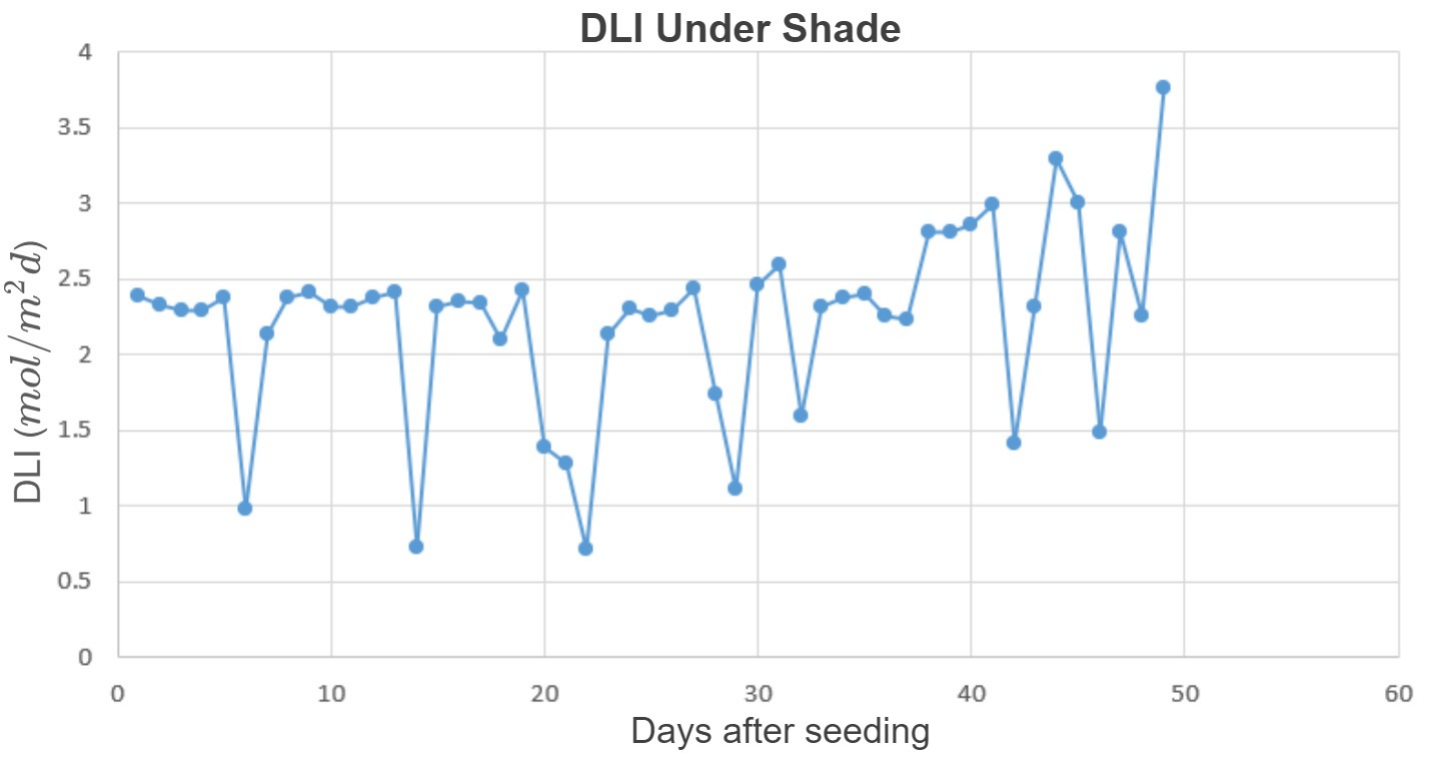
DLI from sunlight under the shade cloth during the first experiment.

**Figure 4:**
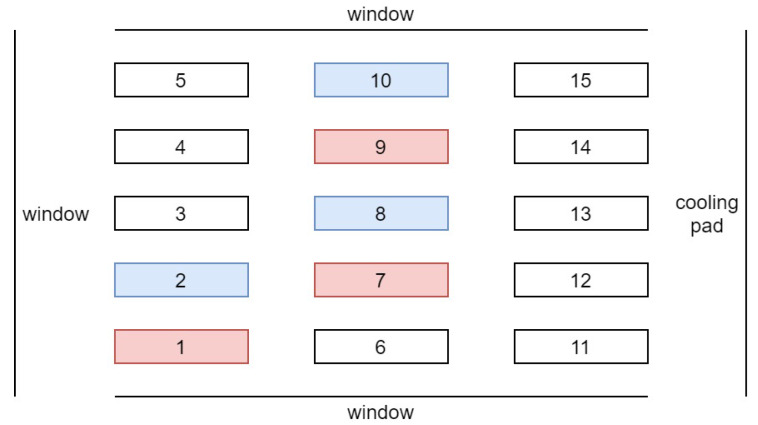
Experimental units in the greenhouse under two different lighting treatments in the first experiment. Plants on the pink benches were grown using the prediction-based lighting method, and those on the blue benches were grown using the heuristic method.

The first experiment ran from 11 December 2020 through 28 January 2021, when sunlight levels were low. The daily minimum temperature, max temperature, minimum vapor pressure deficit (VPD), and maximum VPD inside the greenhouse were 12.4±3.4°C, 22.4±2.8°C, 0.46±0.15kPa, and 1.81±0.63kPa (mean ± SD), respectively. The CO_2_ level was at the ambient level; the average DLI from sunlight under the shade cloth was 2.22 ± 0.6 mol/m${}^{2}$d, and the daily amounts are provided in Figure 3. The setup in Figure 2 was installed, and three lettuce replicates for the heuristic method and three for the proposed optimal method with six LEDs were considered. Figure 4 depicts the greenhouse section in the first experiment. On each bench, 15 plants were grown as 1 experimental unit. This section of the greenhouse was surrounded by windows except for the west wall, where the cooling pad was. Therefore, the benches on the west side may received less sunlight late in the day. It is also possible that the temperatures may have been slightly lower on the west side due to the cooling pad. However, very little cooling was required in winter. Ambient light and temperature can affect plant growth, and the treatments were blocked to account for such effects. Further details are provided in the Results and Discussion Section. Plants were harvested 49 d after seeding, and the proposed method was evaluated in terms of both the electricity cost and plant growth.

**Figure 5:**
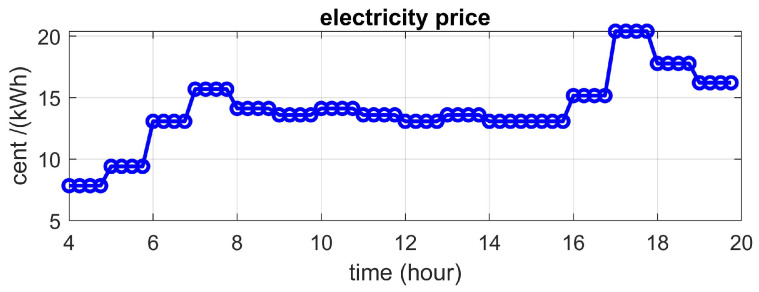
Variable electricity price in cents/kWh used in the first experiment.

For the imaging part, only two cameras were installed above the two benches, one controlled by the heuristic method (Bench Number 8 in Figure 4) and the other one by the prediction-based method (Bench Number 7 in Figure 4). Thus, we used two Raspberry Pis in this experiment, one connected to a camera for taking pictures of one replicate of the heuristic method and another to take pictures of the plant under the prediction-based treatment and to control the output light of all six LED fixtures. During the first experiment, the photoperiod started at 4:30 a.m. and ended at 8:30 p.m. At 8:30 p.m., the LEDs turned off, and the camera automatically took images at 11:30 pm when there was no supplemental light in the greenhouse. We obtained a variable hourly electricity price profile from a website (https://www.ieso.ca/power-data, accessed 1 December 2019) and scaled the numbers to account for the U.S. electricity prices based on government rates. The electricity price profile considered in this experiment is given in Figure 5 and was the same throughout the study.

**Remark** **1.** *The units of f in (1) are $cents\;{\left(kWh\right)}^{-1}\;\mu mol\;m^{-2}\;s^{-1}$; therefore, a conversion factor is needed to convert these units to $cents/m^{2}$. Converting the PPFD of GE LEDs to $kW\;m^{-2}$, $2.9\;\mu mol\;J^{-1}$ (or equivalently $2.9\times 10^{3}\;\mu mol\;{\left(kW\right)}^{-1}\;s^{-1}$) was performed [34]. Assuming the length of each time step (15min or 0.25h), the conversion factor q is defined as:*

(3) $$\begin{array}{c} q=\frac{1}{2.9\times 10^{3}}\times 0.25. \end{array}$$

*Therefore, q×f represents the electricity cost of supplemental lighting with units of $cents/m^{2}$.*

Other than the PCS, the leaf chlorophyll content index (CCI), anthocyanin content index (ACI), specific leaf area (SLA), and shoot dry weight were measured to compare plant growth for the two lighting approaches. The CCI and ACI were measured using chlorophyll and anthocyanin meters (CCM-200 plus and ACM-200 plus; Apogee Instruments, Logan, UT, USA) on the uppermost fully expanded leaves at two different times, once partway through the study and again near the end. Each time, 10 measurements on each experimental unit (group of 15 plants) were collected. The average value of these measurements was considered for each unit. A higher CCI can increase the light absorptance [35], and the anthocyanins in leaves have a protective role against intense light [35].

The SLA was calculated by dividing the leaf area of a plant by the shoot dry weight. For each experimental unit (15 plants), 3 plants were selected, and the total leaf area was measured using a leaf area meter (LI-3100 leaf area meter; LI-COR Biosciences, Lincoln, NE, USA). When drying the plants in the oven, these three plants were placed separately from the other plants. After drying them at 70°C for 7 d, the shoot dry weight of each group of plants was also measured, thereby achieving the SLA per plant. Furthermore, dividing the total dry weight over the number of pots for each unit (15), we obtained the shoot dry weight per plant.

A paired t-test (two-tailed form) was performed for each growth parameter to determine if the two lighting methods had different results. In this test, each replicate was considered to be a pair, and the mean values for the two treatments were compared. If the *p*-value was less than the significance level (here, 0.05), the null hypothesis was rejected, which means there was a significant difference between the two methods.

**Figure 6:**
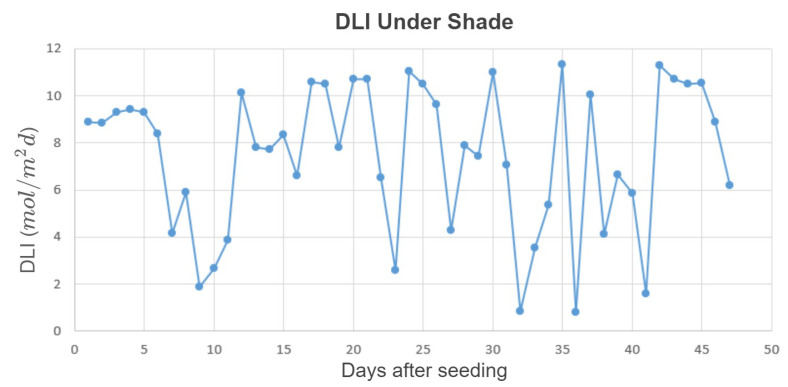
DLI from sunlight under the shade cloth during the second experiment.

The second experiment was conducted using the same crop species in the same place, from 2 April 2021 through 18 May 2021, when sunlight levels were higher than in the first experiment. The minimum temperature, max temperature, minimum vapor pressure deficit (VPD), and maximum VPD inside the greenhouse were 18.7±1.5°C, 25.8±1.5°C, 0.52±0.21kPa, and 1.86±0.75kPa (mean ± SD), respectively. The CO_2_ level was at the ambient level; the average DLI from sunlight under the shade cloth was 7.45 ± 3.11 mol/m${}^{2}$d, and the daily amounts are given in Figure 6. Not only were the sunlight levels much higher than in the first experiment, they were also more variable, thus increasing the potential benefits of an optimized lighting control approach.

**Figure 7:**
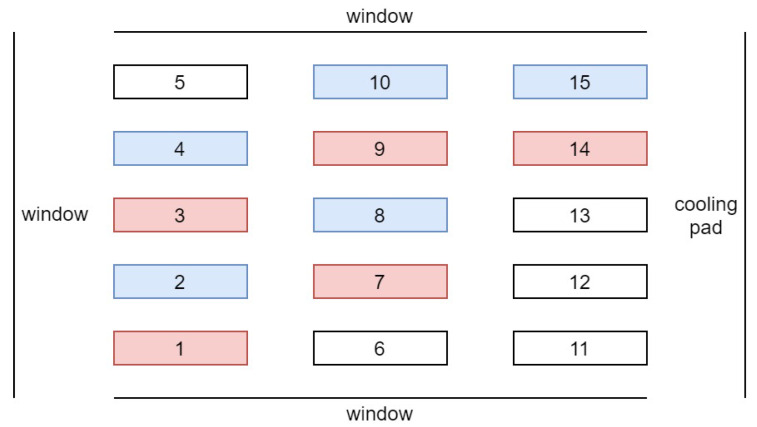
Experimental units in the greenhouse under two different lighting treatments in the second experiment. Pink color is an indicator of the prediction-based lighting method, while blue is for the heuristic method.

This time, we experimented on more replicates, i.e., five replicates for each treatment, which is represented in Figure 7. Plants were harvested 47 d after seeding. The same hardware setup was utilized for this experiment; however, the PCS was monitored for three replicates of each treatment using six Arducam cameras. The cameras were installed above Bench Numbers 1 and 2, 7 and 8, and 14 and 15 (see Figure 7). Moreover, the lighting control started at 5 am every day and ended at 9 pm. This change was made to ensure that the photoperiod fully encompassed the natural photoperiod. Another different factor compared to the first experiment was the use of a fixed electricity price (13.19 cents/kWh), rather than real-time pricing. Considering real-time pricing is advantageous to reduce the electricity cost of lighting. However, for the second experiment, we intended to use the electricity price available to our research greenhouse, which is fixed. Measurements of the growth parameters were repeated for the second experiment, and paired t-tests (two-tailed form) were performed to accept or reject the null hypothesis.

## 3. Results and Discussion

**Figure 8:**
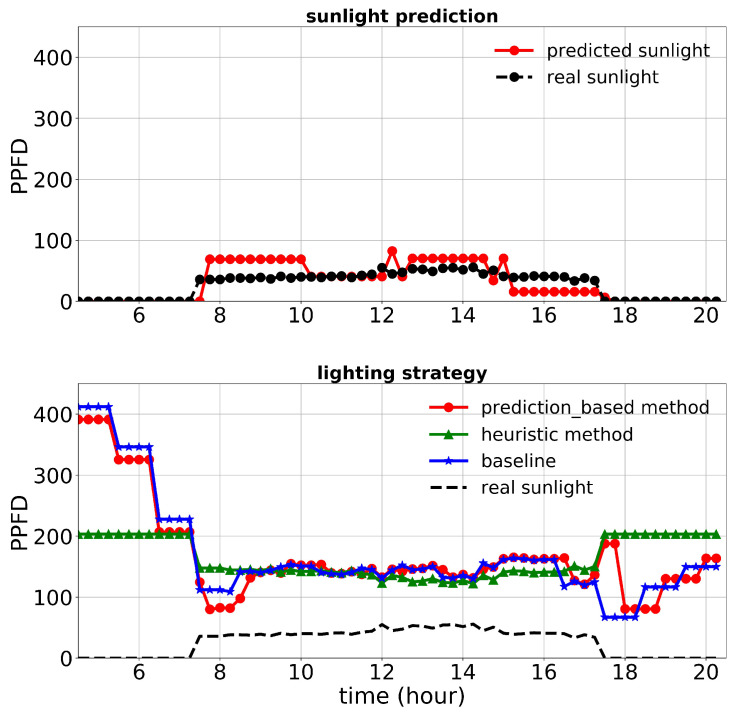
Performance of lighting control strategies and sunlight prediction for December 24th, a day with a low sunlight level during the first experiment.

**Figure 9:**
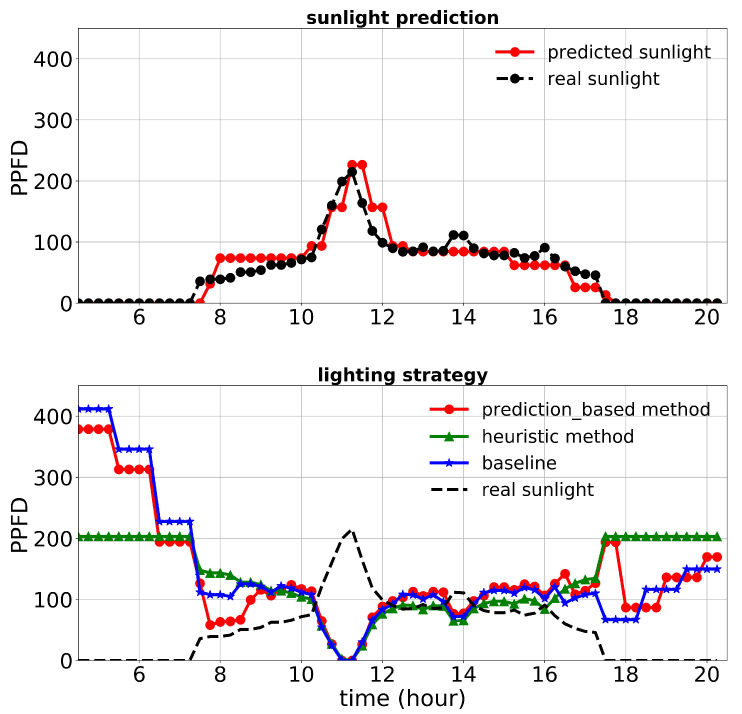
Performance of lighting control strategies and sunlight prediction for January 14th, a day with a moderate sunlight level during the first experiment.

**Figure 10:**
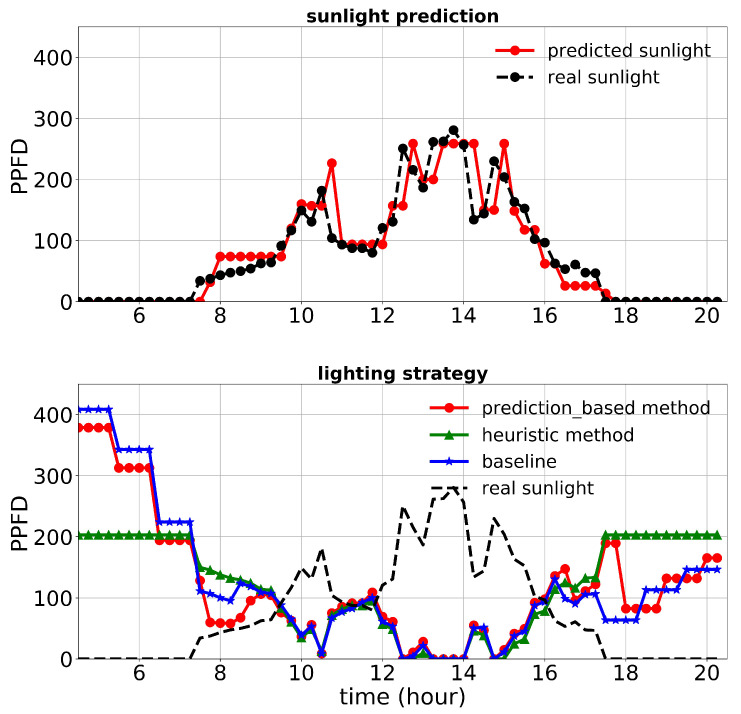
Performance of lighting control strategies and sunlight prediction for January 28th, a day with a high sunlight level during the first experiment.

**Figure 11:**
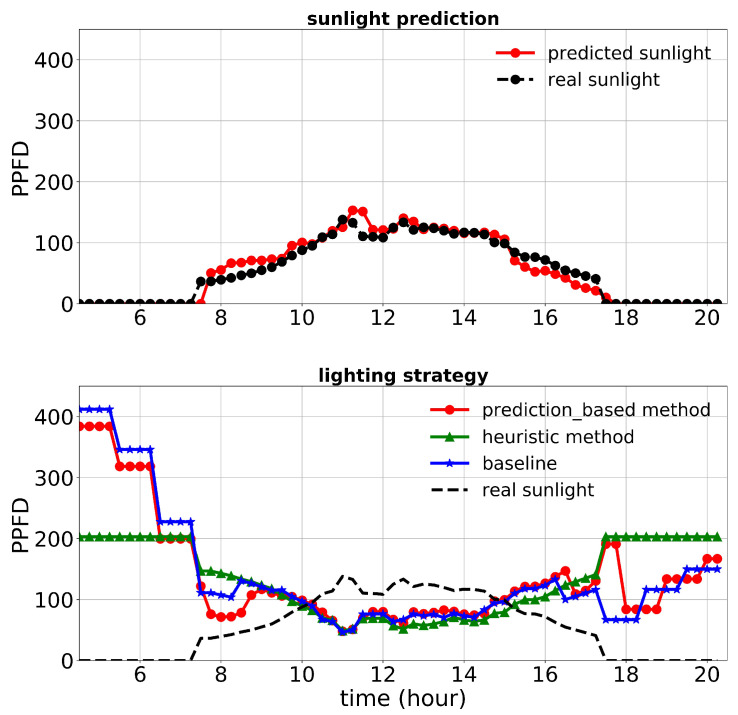
Performance of lighting control strategies and sunlight prediction for the whole period of the first experiment (49 d) as an average.

First, we discuss the results of the first experiment. The lighting control performance and sunlight prediction throughout different days with different sunlight levels are shown in Figure 8, Figure 9 and Figure 10. Figure 8 is representative of a rainy day with very low sunlight irradiance. January 14th’s irradiance was close to a day with regular sunlight during that period, and Jan 28th had a relatively high sunlight irradiance (presented in Figure 9 and Figure 10, respectively). The cost increase on each day compared to the baseline was calculated and shown in Table 3. Moreover, the average of actual sunlight, its prediction, and supplemental light for different methods were calculated throughout this experiment and displayed in Figure 11. Based on the results in Table 3, the cumulative cost increase for the heuristic method was 5.45%, while for the prediction-based method, it was 1.06% compared to the baseline. Therefore, our prediction-based lighting approach showed about a 4.16% electricity cost reduction compared to the heuristic method throughout the first experiment.

In the baseline approach, perfect prior knowledge of sunlight throughout the day is assumed, which is not practical and only represents a theoretical optimal scenario. A predictive model is not able to predict sunlight with 100% accuracy; therefore, the sunlight information in the baseline approach is always more accurate than that in the proposed method. In other words, the baseline is an ideal optimal scenario with the least electricity cost, which is not practical. However, the prediction-based method is optimal and practical, which resulted in a very close solution to the baseline (the global optimal solution of the lighting problem). The heuristic lighting strategy generally provided more light than the minimum DPI and resulted in extra supplemental lighting cost; thus, it is not optimal.

**Figure 12:**
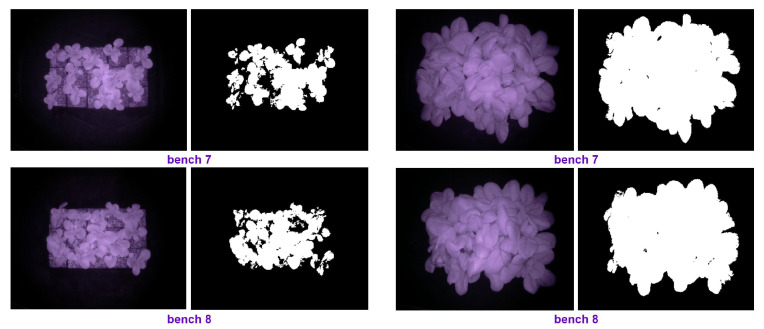
The original and the processed images on Day 32 and the last day of the first experiment.

Some of the images taken by our setup (see Figure 1) are shown in Figure 12. The top images were taken of the plants under prediction-based lighting, and the bottom images were taken of the plants under the heuristic lighting method. The left pairs in Figure 12 were captured 32 d after seeding (January 11th), and the right pairs were captured at the end of the experiment (January 28th). Arducam took the left images of each pair at night in darkness, and then, the images were transferred to a computer through the VNC viewer. Hence, we could access the images remotely and in real time via WiFi. Using the PlantCV package, the images were analyzed, and the PCS was measured by thresholding, thereby resulting in the right images of each pair in Figure 12.

The results of the statistical analysis on all growth parameters are provided in Table 4. The *p*-value for all parameters was greater than 0.05; consequently, the means of the two treatments were almost equal for each growth parameter (especially shoot dry weight, which was the most important growth parameter). Therefore, the proposed approach did not affect the plant growth adversely or positively. We calculated another parameter, which was the total dry weight divided by the total electricity cost (in cents/m${}^{2}$), for each approach. This parameter includes both cost and growth information, which for the prediction-based method was 0.0797 (g/cent) and for the heuristic method was 0.0738 (g/cent), a 7.4% reduction. Hence, the proposed optimal lighting approach resulted in a reduction of the electricity cost and did not affect plant growth adversely.

**Figure 13:**
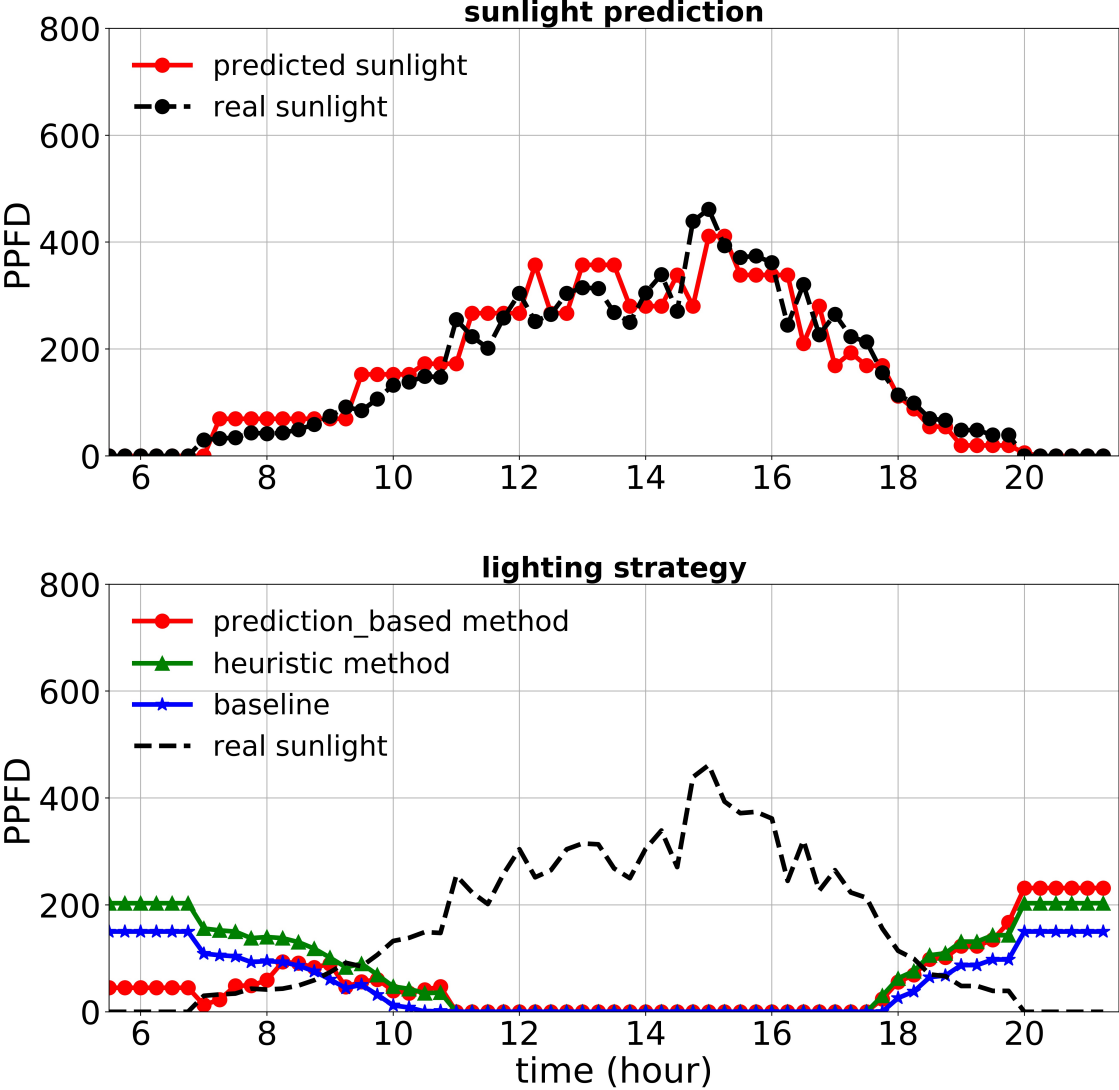
Performance of lighting control strategies and sunlight prediction for April 15th, a day with a low sunlight level during the second experiment.

**Figure 14:**
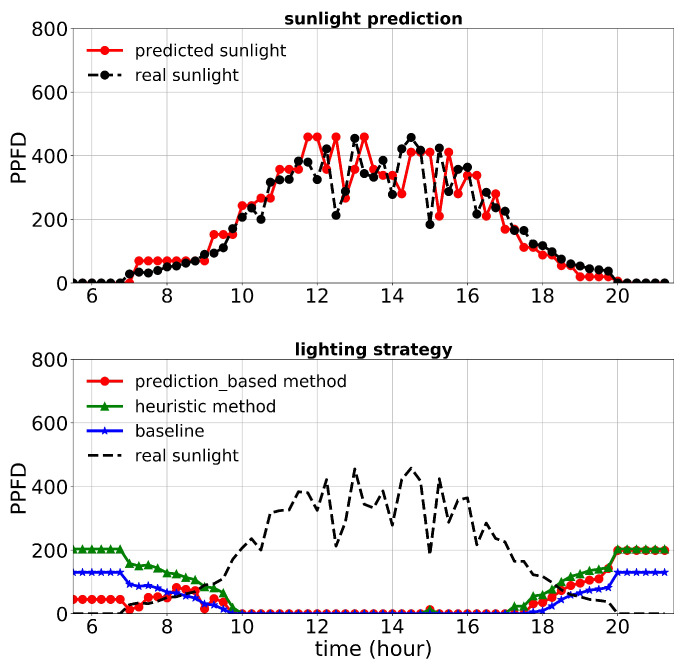
Performance of lighting control strategies and sunlight prediction for April 7th, a day with a moderate sunlight level during the second experiment.

**Figure 15:**
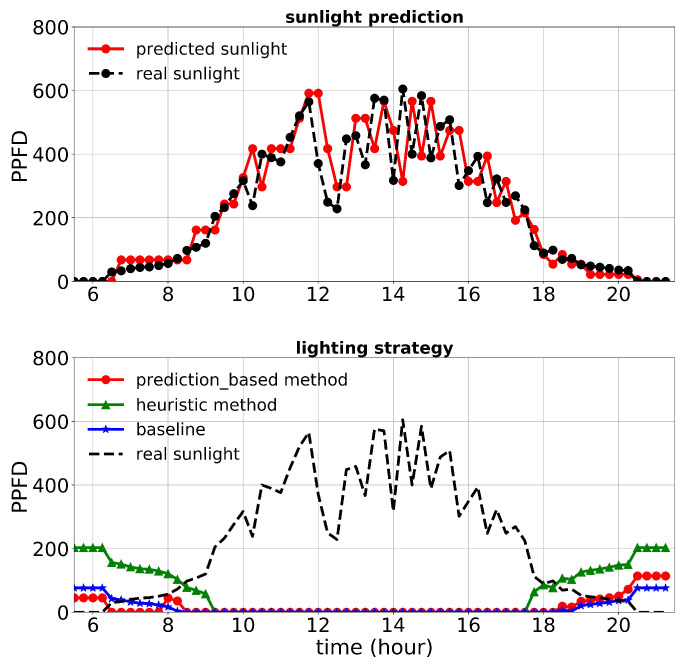
Performance of lighting control strategies and sunlight prediction for May 6th, a day with a high sunlight level during the second experiment.

**Figure 16:**
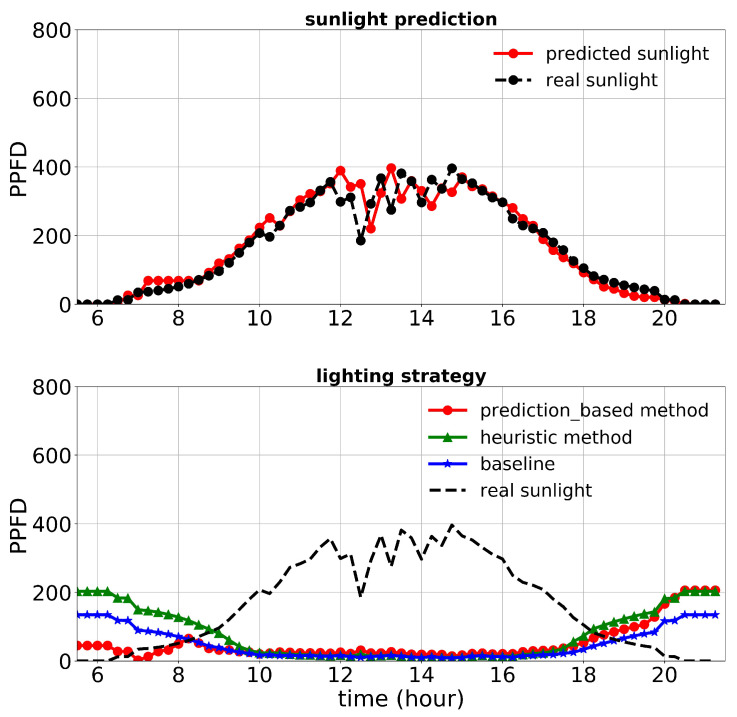
Performance of lighting control strategies and sunlight prediction for the whole period of the second experiment (47 d) as an average.

Figure 13, Figure 14 and Figure 15 show the performance of the lighting strategies and sunlight prediction throughout different days with different sunlight levels in the second experiment. Figure 13 is representative of rainy days with low sunlight irradiance compared to the mean irradiance in April. April 7th irradiance was close to a day with regular sunlight during that period, and May 6th had high sunlight irradiance (presented in Figure 14 and Figure 15, respectively). The cost increase in each day compared to the baseline was calculated and shown in Table 5. Moreover, the average of actual sunlight, its prediction, and supplemental light for different methods were calculated throughout this experiment and displayed in Figure 16. Based on the results in Table 5, the cumulative cost increase for the heuristic method was 62.86%, while for the prediction-based method, it was 7.74% compared to the baseline. Our prediction-based lighting approach showed about a 33.85% electricity cost reduction compared to the heuristic method throughout the second experiment.

**Figure 17:**
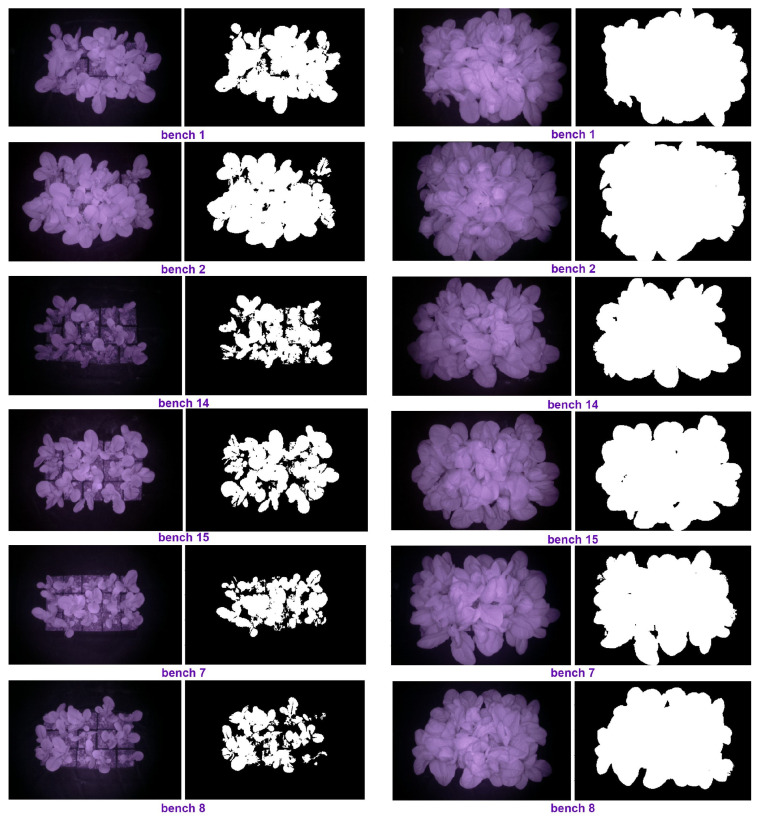
The original and the processed images on Day 33 and the last day of the second experiment.

**Figure 18:**
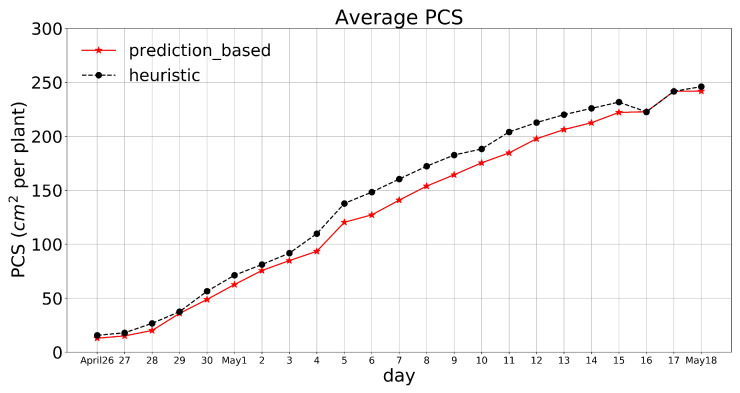
Average PCS for the two treatments during the last 23 d of the second experiment.

Some of the images taken by our setup (see Figure 1) during the second experiment are illustrated in Figure 17. The left pairs were captured 33 d after seeding (May 4th), and the right pairs were captured at the end of the experiment (May 18th). The PCS was measured using PlantCV package, and we monitored the plant growth remotely via WiFi in real-time, the same method as the first experiment. The average of the PCS for those replicates that had a camera was measured for each treatment and shown in Figure 18.

Other than the lighting approach, different temperatures and ambient light in the greenhouse had at most minor effects on our results, since our treatments were blocked to account for east to west environmental gradients within the greenhouse.

The results of the statistical analysis on the growth parameters for the second experiment are provided in Table 6. The *p*-value for all parameters was greater than 0.05, except for the SLA (t(4)=3.37,p=0.03). The SLA was greater for the proposed strategy (μ=131.24,σ=8.1cm${}^{2}$/g per plant), compared to the heuristic method (μ=116.69,σ=7.34 cm${}^{2}$/g per plant). However, this difference was not necessarily due to a true biological effect. It was more likely due to a Type I error. Thus, the statistical analysis did not show meaningful differences in plant growth between the two treatments. The total shoot dry weight divided by the total cost for the prediction-based method was 0.2166 (g/cent), while for the heuristic method, it was 0.1475 (g/cent), 32% lower. Hence, the proposed strategy resulted in a higher efficiency than the heuristic method in terms of electricity cost together with plant growth.

Both experiments validated that the prediction-based method can reduce cost while maintaining plant growth. As shown in Figure 3 and Figure 6, the sunlight levels were much lower during the first study compared to the second study; therefore, more supplemental light was provided to reach the minimum DPI during the first study, which resulted in a higher electricity cost (for both approaches). Since the dry weight at the end of the experiments was almost the same, the total dry weight/total cost was lower for the first study. The difference in the DLI from sunlight also affected the electricity cost savings of the proposed method for the two experiments. During the first study, the DLI was very low, and that resulted in providing so much supplemental light to satisfy the light requirements of the plants with both lighting approaches (the heuristic and the proposed method). The heuristic method provided the greatest benefits when the light levels are variable, both in the short term (15 min) and longer term (day to day). Thus, there was not a huge difference in the cost of the lighting methods. However, during the second study, the DLI levels were much higher, and much less supplemental lighting was needed to reach the minimum lighting requirement. Hence, the proposed method reduced the cost by a higher percentage in the second study (33.85% compared to 4.16%).

The cost profile in the second experiment was a fixed price, since we wanted to consider the actual conditions in our research greenhouse. The simulation results in our previous work [25] showed that the proposed method with variable electricity pricing contributed to a cost reduction in different months of the year. If we used a variable cost profile for the second study, as well as the first one, the cost reduction in the second study would have been even higher.

The proposed strategy for controlling supplemental lighting significantly differs from the previous approaches. Most of the previous approaches that were mentioned in the Introduction Section are rule-based methods and do not use sunlight prediction [15,17,22]. Among all the methods proposed for HID lamps, the DynaLight system and DynaLight IND are the most efficient ones since they use weather forecast, a photosynthesis model, and a variable electricity profile. However, these methods are still not optimal because providing the weather forecasts twice daily is not enough to obtain the optimal lighting strategy, and for the improved version of this system (DynaLight IND), practical experiments should be conducted to validate its efficiency [20,21]. On the other hand, our proposed method was evaluated through both simulations and practical experiments.

A study on an adaptive control approach for LEDs [15] showed that a method similar to the heuristic method reduces the cost of supplemental lighting, compared to two other rule-based methods. In the present paper, we concluded that the prediction-based approach reduced the cost significantly compared to the heuristic method. Therefore, our strategy outperformed the other methods by taking advantage of the sunlight predictive model, the minimum DPI requirement, and variable electricity pricing. The shortcoming of our method is that the sunlight predictive model uses the mean value of the historical data at the beginning of the day, and for rainy days with too low sunlight levels, this may result in overestimating sunlight. Therefore, this approach may not perform as expected in this case.

There has been much research on growing lettuce with 24 h photoperiods [36]. Nonetheless, this practice has not (yet) been adopted by the industry, where photoperiods of 16–20 h are more commonly used. To make our research relevant to the greenhouse industry, we decided to use a photoperiod that is commonly used in commercial greenhouses. The energy savings that can be realized may indeed depend on the photoperiod that is used. Our lighting optimization protocol was formulated in such a way that any photoperiod can be used, and it is not specific to a 16 h photoperiod.

Finally, we note that lighting can provide a substantial amount of heat to a greenhouse; but the effects of more efficient lighting methods and greenhouse heating requirements are often ignored. A major challenge is that the interaction between lighting and heating is location specific and requires advanced models to estimate [37]. We thus opted to focus solely on lighting costs in this study.

## 4. Conclusions

In this paper, a new optimal approach was developed and implemented for supplemental lighting control in greenhouse environments using IoT technology. This method solves an optimization problem to satisfy plant light needs using Markov-based sunlight prediction and variable electricity pricing. Two experimental studies during two different seasons in the same greenhouse were conducted, where the proposed lighting approach was validated in terms of electricity cost reduction (4.16% reduction during the winter study and 33.85% during the spring study) while maintaining plant growth.

## Figures and Tables

**Table 1:** Model and optimization parameters used in this work.

Variable	Value	Variable	Value
*a*	121 µmol m${}^{-2}$ s${}^{-1}$	m	900 s
*D*	3 mol m${}^{-2}$ d${}^{-1}$	*T*	64
${\bar{U}}_{LED}$	86.21 µmol m${}^{-2}$ s${}^{-1}$	*k*	0.00277

**Table 2:** Main materials used in our experimental setup.

Item	Details
plant	“Green Towers” lettuce
lighting source	GE Arize${}^{\mathrm{TM}}$ element L1000 LED grow light bar
light sensor	SQ-500-SS
microprocessor	Raspberry Pi 3 Model B
signal-conditioning circuit	TL081 op-amp, 24V DC power supply adapter, resistors, and capacitors
ADC	ADS1115
camera	Arducam automatic day/night camera module (model number: B003503)

**Table 3:** Cost of lighting control strategies in $/m${}^{2}$ per day for different days during the first experiment.

Day	Baseline Cost	Prediction-Based Method Cost	Heuristic Method Cost
		Increase Compared to Baseline	Increase Compared to Baseline
December 24	0.39	0.0027 (0.7%)	0.0174 (4.46%)
January 14	0.33	0.0040 (1.21%)	0.0178 (5.39%)
January 28	0.27	0.0038 (1.41%)	0.0286 (10.59%)
cumulative cost	16.14	0.17 (1.05%)	0.88 (5.45%)

**Table 4:** Results of the paired *t*-test on the growth parameters during the first experiment.

Measurement	Mean (Prediction-Based)	Mean (Heuristic)	Standard Deviation (Prediction-Based)	Standard Deviation (Heuristic)	Standard Error (Prediction-Based)	Standard Error (Heuristic)	*p*-Value	*t*-Value
CCI/January 11th	16.36	17.1	2.84	1.68	1.64	0.97	0.40	1.06
CCI/January 28th	24.06	22.93	1.94	1.42	1.12	0.82	0.33	1.26
ACI/January 11th	4.86	4.91	0.45	0.13	0.26	0.08	0.82	0.25
ACI/January 28th	5.56	5.58	0.64	0.17	0.37	0.1	0.96	0.06
SLA per plant (cm${}^{2}$/g)	114.17	117.67	3.66	11.7	2.11	6.76	0.54	0.73
shoot dry weight per plant (g)	2.89	2.79	0.41	0.36	0.24	0.21	0.8	0.28

**Table 5:** Cost of lighting control strategies in $/m${}^{2}$ per day for different days during the second experiment.

Day	Baseline Cost	Prediction-Based Method Cost Increase Compared to Baseline	Heuristic Method Cost Increase Compared to Baseline
April 7	0.16	0.0085 (5.31%)	0.14 (87.5%)
April 15	0.21	0.0135 (6.43%)	0.11 (52.38%)
May 6	0.06	0.0015 (2.5%)	0.21 (350%)
cumulative cost	9.49	0.73 (7.69%)	5.96 (62.8%)

**Table 6:** Results of the paired *t*-test on the growth parameters during the second experiment.

Measurement	Mean (Prediction-Based)	Mean (Heuristic)	Standard Deviation (Prediction-Based)	Standard Deviation (Heuristic)	Standard Error (Prediction-Based)	Standard Error (Heuristic)	*p*-Value	*t*-Value
CCI/May 7th	9.81	10.54	0.80	0.73	0.36	0.33	0.19	1.98
CCI/May 18th	12.19	11.80	1.40	0.76	0.63	0.34	0.62	0.54
ACI/May 7th	3.63	3.67	0.22	0.14	0.1	0.06	0.77	0.32
ACI/May 18th	4.1	4.02	0.16	0.25	0.07	0.11	0.49	0.75
SLA per plant (cm${}^{2}$/g)	131.24	116.69	8.1	7.34	3.62	3.28	0.03	3.37
PCS per plant (cm${}^{2}$)	241.93	246.06	14.42	32.99	8.33	19.05	0.78	0.33
shoot dry weight per plant (g)	2.95	3.04	0.45	0.42	0.2	0.19	0.06	2.64

## Data Availability

Sunlight data that were used to develop the predictive model are available at https://www.nrel.gov/grid/solar-resource/confrrm.html (accessed on 1 December 2019). The code developed for implementing the lighting strategy can be found here: https://github.com/velnilab/optimal-lighting (released 19 November 2021).

## Abbreviations

The following abbreviations are used in this manuscript:

ACI	Anthocyanin content index
ADC	Analog-to-digital converter
CCI	Leaf chlorophyll content index
CEA	Controlled environment agriculture
DLI	Daily light integral
DPI	Daily photochemical integral
DSL	Domain-Specific Language
ETR	Electron transport rate
FPGA	Field programmable gate array
GA	Genetic algorithm
HCI	Human–computer interaction
HID	High-intensity discharge
IoT	Internet of Things
IR	Infrared
LASSI	Light and shade system implementation
LED	Light-emitting diode
PCS	Projected canopy size
PPFD	Photosynthetic photon flux density
PWM	Pulse-width modulation
RFID	Radio-frequency identification
RL	Reinforcement learning
SLA	Specific leaf area
UAV	Unmanned aerial vehicle
VPD	Vapor pressure deficit
WSN	Wireless sensor network

## References

[1] Alexandratos N., Bruinsma J. (2012). World Agriculture towards 2030/2050: The 2012 Revision.

[2] Khanna A., Kaur S. (2019). Evolution of Internet of Things (IoT) and its significant impact in the field of Precision Agriculture. Comput. Electron. Agric..

[3] Elijah O., Rahman T.A., Orikumhi I., Leow C.Y., Hindia M.N. (2018). An overview of Internet of Things (IoT) and data analytics in agriculture: Benefits and challenges. IEEE Internet Things J..

[4] Manrique J.A., Rueda-Rueda J.S., Portocarrero J.M. Contrasting internet of things and wireless sensor network from a conceptual overview. Proceedings of the 2016 IEEE international conference on Internet of Things (iThings) and IEEE Green Computing and Communications (GreenCom) and IEEE Cyber, Physical and Social Computing (CPSCom) and IEEE SMART data (SmartData).

[5] Zhao J.C., Zhang J.F., Feng Y., Guo J.X. The study and application of the IoT technology in agriculture. Proceedings of the 2010 3rd International Conference on Computer Science and Information Technology.

[6] Odema M., Adly I., Wahba A., Ragai H. Smart aquaponics system for industrial Internet of Things (IIoT). Proceedings of the International Conference on Advanced Intelligent Systems and Informatics.

[7] Yu Z., Xugang L., Xue G., Dan L. (2014). IoT forest environmental factors collection platform based on ZIGBEE. Cybern. Inf. Technol..

[8] Shinde T.A., Prasad J.R. (2017). IoT based animal health monitoring with naive Bayes classification. Int. J. Emerg. Trends Technol..

[9] Tripicchio P., Satler M., Dabisias G., Ruffaldi E., Avizzano C.A. Towards smart farming and sustainable agriculture with drones. Proceedings of the 2015 International Conference on Intelligent Environments.

[10] Mainetti L., Mele F., Patrono L., Simone F., Stefanizzi M.L., Vergallo R. (2013). An RFID-based tracing and tracking system for the fresh vegetables supply chain. Int. J. Antennas Propag..

[11] Lerdsuwan P., Phunchongharn P. An energy-efficient transmission framework for IoT monitoring systems in precision agriculture. Proceedings of the International Conference on Information Science and Applications.

[12] Liu H., Meng Z., Cui S. A wireless sensor network prototype for environmental monitoring in greenhouses. Proceedings of the 2007 International Conference on Wireless Communications, Networking and Mobile Computing.

[13] Zhang Q., Yang X.L., Zhou Y.M., Wang L.R., Guo X.S. (2007). A wireless solution for greenhouse monitoring and control system based on ZigBee technology. J. Zhejiang-Univ.-Sci. A.

[14] Pekoslawski B., Krasinski P., Siedlecki M., Napieralski A. Autonomous wireless sensor network for greenhouse environmental conditions monitoring. Proceedings of the 20th International Conference Mixed Design of Integrated Circuits and Systems-MIXDES 2013.

[15] van Iersel M.W., Gianino D. (2017). An adaptive control approach for light-emitting diode lights can reduce the energy costs of supplemental lighting in greenhouses. HortScience.

[16] Narra P., Zinger D.S. An effective LED dimming approach. Proceedings of the Conference Record of the 2004 IEEE Industry Applications Conference, 2004. 39th IAS Annual Meeting.

[17] Carrier M., Gosselin A., Gauthier L. (1994). Description of a crop growth model for the management of supplemental lighting in greenhouses. HortTechnology.

[18] Albright L., Both A.J., Chiu A. (2000). Controlling greenhouse light to a consistent daily integral. Trans. ASAE.

[19] Seginer I., Albright L.D., Ioslovich I. (2006). Improved strategy for a constant daily light integral in greenhouses. Biosyst. Eng..

[20] Mærsk-Møller H.M., Jørgensen B.N. A software product line for energy-efficient control of supplementary lighting in greenhouses. Proceedings of the The International Conference on Green Computing.

[21] Qu Y., Clausen A., Jørgensen B.N. (2021). A multi-objective optimization platform for artificial lighting system in commercial greenhouses. Energy Informatics.

[22] Pinho P., Hytönen T., Rantanen M., Elomaa P., Halonen L. (2013). Dynamic control of supplemental lighting intensity in a greenhouse environment. Light. Res. Technol..

[23] Weaver G.M., van Iersel M.W., Velni J.M. (2019). A photochemistry-based method for optimising greenhouse supplemental light intensity. Biosyst. Eng..

[24] Schwend T., Beck M., Prucker D., Peisl S., Mempel H. (2016). Test of a PAR sensor-based, dynamic regulation of LED lighting in greenhouse cultivation of Helianthus annuus. Eur. J. Hortic. Sci..

[25] Mosharafian S., Afzali S., Weaver G.M., van Iersel M., Velni J.M. (2021). Optimal lighting control in greenhouse by incorporating sunlight prediction. Comput. Electron. Agric..

[26] Afzali S., Mosharafian S., van Iersel M.W., Velni J.M. Optimal Lighting Control in Greenhouses Equipped with High-intensity Discharge Lamps Using Reinforcement Learning. Proceedings of the 2021 American Control Conference (ACC).

[27] Elkins C., van Iersel M.W. (2020). Longer photoperiods with the same daily light integral increase daily electron transport through photosystem II in lettuce. Plants.

[28] Weaver G., van Iersel M.W. (2020). Longer photoperiods with adaptive lighting control can improve growth of greenhouse-grown ‘Little Gem’ lettuce (Lactuca sativa). HortScience.

[29] Diamond S., Boyd S. (2016). CVXPY: A Python-embedded modeling language for convex optimization. J. Mach. Learn. Res..

[30] Agrawal A., Verschueren R., Diamond S., Boyd S. (2018). A rewriting system for convex optimization problems. J. Control. Decis..

[31] NREL National Renewable Energy Laboratory (NREL). https://www.nrel.gov/grid/solar-resource/confrrm.html.

[32] Zabeltitz C. (2011). Light Transmittance of Greenhouses. Integrated Greenhouse Systems for Mild Climates.

[33] Gehan M.A., Fahlgren N., Abbasi A., Berry J.C., Callen S.T., Chavez L., Doust A.N., Feldman M.J., Gilbert K.B., Hodge J.G. (2017). PlantCV v2: Image analysis software for high-throughput plant phenotyping. PeerJ.

[34] Arize GE Arize Element L1000 Specification Sheet. https://images.salsify.com/images/rf2bqhdet0yfnygoabjh/HORT100-Arize-Element-L1000-Spec-Sheet.pdf.

[35] Jayalath T.C., van Iersel M.W. (2021). Canopy Size and Light Use Efficiency Explain Growth Differences between Lettuce and Mizuna in Vertical Farms. Plants.

[36] Koontz H., Prince R. (1986). Effect of 16 and 24 h daily radiation (light) on lettuce growth. Hort Sci. Publ. Am. Soc. Hortic. Sci..

[37] Katzin D., Marcelis L.F., van Mourik S. (2021). Energy savings in greenhouses by transition from high-pressure sodium to LED lighting. Appl. Energy.

